# New data on nutrient composition in large selection of commercially available seafood products and its impact on micronutrient intake

**DOI:** 10.29219/fnr.v63.3573

**Published:** 2019-07-08

**Authors:** Inger Aakre, Synnøve Næss, Marian Kjellevold, Maria Wik Markhus, Anita Røyneberg Alvheim, Jorån Østerholt Dalane, Ellen Kielland, Lisbeth Dahl

**Affiliations:** 1 Food Security and Nutrition, Institute of Marine Research, Bergen, Norway; 2 Food Department, Norwegian Food Safety Authorities, Brumunddal, Norway

**Keywords:** nutrients, food composition, seafood, seafood products, micronutrients

## Abstract

**Background:**

Most foods, including seafood, undergo some sort of processing as an integrated part of the global food industry. The degree of processing depends on the type of product produced. Processed seafood products are an important part of the diet; thus, knowledge of nutrient content in seafood products is of great importance.

**Objective:**

The aim of this study was to describe the content of selected nutrients in commercially available and market representative seafood products purchased from 3 different years.

**Methods:**

Seafood products from 2015 (*n* = 16), 2017 (*n* = 35), and 2018 (*n* = 35) were analyzed as composite samples for macro- and micronutrients using accredited methods at the Institute of Marine Research in Norway.

**Results:**

This study confirms that seafood products are good sources of several key nutrients, such as eicosapentaenoic acid (EPA) and docosahexaenoic acid (DHA), vitamin D, vitamin B12, iodine, and selenium. Fatty fish products had the highest content of EPA, DHA, and vitamin D, while lean fish products had the highest content of vitamin B12 and minerals. However, some lean fish products, such as one portion of fish au gratin or fish cakes, also proved as good sources of EPA, DHA, and vitamin D, and contributed substantially to the recommended intake. Variations in nutrients were seen both within the same product category and between the same product category from different years.

**Conclusions:**

These data give valuable insights into seafood products as a source of essential micronutrients and highlight the importance of these products for nutrition and health.

## Popular scientific summary

- Processed seafood products contain significant amounts of essential nutrients.
- Not only fatty fish products but also some lean fish products, such as fish au gratin and fish cakes, may be good sources of EPA, DHA, and vitamin D.
- These results contribute to food composition data and nutrient intake estimations.
- Monitoring of nutrient composition in seafood products is important as they are in continuous development.

Food composition data are an essential basic tool for nutrition research, such as evaluating dietary intake in individuals and population groups. Having available food composition data are important for estimating nutrient intake, assess nutrient requirements, develop dietary guidelines, and calculate nutrient values used in labeling, and for making nutrition, food security, and agricultural policies (1–4). Biodiversity, local food traditions, and preferences need to be considered when developing national food databases (3).

Fish is a good source of several key nutrients, such as eicosapentaenoic acid (EPA) and docosahexaenoic acid (DHA), vitamin D, vitamin B12, iodine, and selenium (5–7). It is also a good source of highly bioavailable proteins, which make it unique in a nutritional context (8). Fish enhances the bioavailability of minerals like zinc and iron from cereal-based foods and is therefore important in a healthy and balanced diet (9–12). Thus, including even small amounts of fish in the diet may enhance the overall micronutrient intake and bioavailability.

Nutrient content varies within and between fish species depending on edible parts, habitat, region, and seasons (13). Typically, lean fish contains higher levels of iodine (15) whereas fatty fish contains higher levels of vitamin D, EPA, and DHA (14). We have previously reported large variation in iodine levels in lean fish, ranging from 22 to 720 μg/100 g in Atlantic cod (*Gadus morhua*) (15).

Today, most foods, including seafood, undergo some sort of processing as an integrated part of the global food industry. The degree of processing depends on the type of product produced. In Norway, there is a trend that consumers prefer easily prepared seafood and fish products (16). There is a great variety of processed seafood products on the Norwegian market, which include fish farce products (fish cake/burger and fish pudding), mixed products (mackerel in tomato sauce, fish au gratin, caviar, and panned fish), and preserved fish fillet (i.e. smoked fish). In recent years, there has been a tendency of an increased fish content in seafood products on the Norwegian market (17). As processed seafood products are a significant part of the diet of Norwegian consumers (18), the knowledge of nutrient concentration in processed seafood products is important for food and nutrition security.

The aim of this study was to describe the concentration of selected nutrients in a large sample of commercially available seafood products purchased between 2015 and 2018 in Norway, using analytic data.

## Methods

### Sample management

The list of products to be analyzed was determined using detailed product data delivered by Nielsen Norge, supplemented by the market share reports of Market Trends Norway for 2015, 2017, and 2018 from Nielsen Norge. In order to make a representative list of products, best-selling products, products from several brands, store brand products, and low-price products were included. Depending on the product assortment of the Norwegian market in 2015–2018, 1–7 products from each product type (e.g. fish cakes) were selected. The products were analyzed as composite samples consisting of three different batches and were homogenized into one composite sample. When several units were present in one package, minimum three units (e.g. fish cakes) were included from each batch.

The seafood products were purchased from grocery stores either as chilled or as frozen products. The grocery stores were located in Bergen. Due to difficulties in finding one of the samples in Bergen, two sub-samples were purchased from a grocery store in Oslo. After being purchased, the sub-samples were immediately transported to the laboratory at the Institute of Marine Research (IMR), Bergen and stored in a freezer at −80°C until sample preparation.

### Determination of nutrients in the composite samples

All composite samples from each year were analyzed for total protein, total fat, sum saturated fatty acids (SFA), sum monounsaturated fatty acids (MUFA), sum polyunsaturated fatty acids (PUFA), sum n-3, sum n-6, EPA, and DHA. In 2015, vitamin A1, vitamin B12, vitamin D3, iodine, selenium, calcium, zinc, iron, and sodium were analyzed. Due to financial reasons, only vitamin D3, iodine, selenium, zinc, iron, calcium, and sodium were analyzed in addition to protein, fat, and fatty acids in 2017, and vitamin D3, iodine, selenium, calcium, and sodium in 2018. All analyses were performed at the IMR at laboratories using accredited methods with NS-EN ISO/IEC 17025 standards.

All samples were homogenized, freeze-dried, and pulverized before analyses. The dry matter was carried out in accordance with the AOAC 930.15 method. Fat was extracted with ethyl acetate and filtered before the solvent was evaporated, and the fat residue was weighted. The method is accredited in accordance with ISO-EN 17025 and is registered as a Norwegian standard, NS 9402 (Norwegian Standard, 1994). Protein (crude protein) was determined by burning the material in pure oxygen in a combustion tube at 830°C. Nitrogen (N) was detected with a thermal conductivity detector (TCD), and the content of nitrogen was calculated from the area of the peak and by calibration coefficient. The protein content was calculated from an estimated average of 16% N per 100 g protein, and the following formula was used: N g/100 g × 6.25 = protein g/100 g. The method is accredited according to AOAC 992.15 (1995). Fatty acid composition was determined by gas chromatography (GC) after extraction with chloroform:methanol using 19:0 methyl ester as an internal standard. The fatty acids were identified by retention time using standard mixtures of methyl esters (Nu_Check, MN, USA), and the fatty acids were calculated using an integrator (Chromelon 6.8+, Dionex Corporation, CA, USA), connected to the GC. The sample for determination of vitamin A1 (sum all trans-retinol and 13-, 11-, 9 cis retinol) was saponified, and the unsaponifiable material was extracted. Vitamin A was determined by ultra-performance liquid chromatography (UPLC) (normal phase) using the PDA detector (Photo Diode Array). The retinol content was calculated by external calibration (standard curve). The method is validated and accredited, and is based on CEN pr EN 12823-1 (1999), Foodstuffs - Determination of vitamin A by high-performance liquid chromatography (HPLC) - Part 1: Measurement of all trans retinol and 13-cis retinol. The sample for determination of vitamin D was saponified, and the unsaponifiable material was extracted and then purified in a preparative HPLC column. The fraction containing D2 (ergocalciferol) and D3 (cholecalciferol) was pooled (normal phase). This fraction was injected on an analytic HPLC column (reverse phase). Vitamin D3/D2 was determined by ultraviolet (UV) detector at 265 nm. The content of vitamin D3 was calculated using internal standard (vitamin D2). The method is validated and accredited, and is based on CEN pr EN 12821 (1999), Foodstuffs - Determination of vitamin D by high performance liquid chromatography - Measurement of cholecalciferol (D3) and ergocalciferol (D2). Vitamin B12 was released from the sample by extraction (autoclaving in acetate buffer) and mixed with growth medium, added to the microorganism (Lactobacillus delbrueckii-ATCC 4797), and incubated at 37°C for 22 h. The vitamin content was calculated by comparing the growth of the organism in the unknown samples with the growth of the organism in known standard concentrations with turbidimetric reading (Optical Density, OD, v/575 nm). The concentrations of selenium, zinc and iron were determined by Inductively Coupled Plasma-Mass Spectrometry (iCapQ ICPMS, ThermoFisher Scientific, Waltham, MA, USA) equipped with an autosampler (FAST SC-4Q DX, Elemental Scientific, Omaha, NE, USA), after wet digestion in a microwave oven as described by Julshamn et al. 2007 with some modifications (19). The concentrations were determined using an external calibration (standard curve). The method is accredited according to ISO 17025 for selenium and zinc and unaccredited for iron. For the determination of iodine, the sample was microwave digested with tetramethylammonium hydroxide (TMAH) before ICP-MS analysis. The method is accredited according to ISO 17025. The concentration of calcium and sodium were determined by ICPMS after the samples were decomposed using concentrated and extra pure nitric acid and concentrated hydrogen peroxide in the microwave. The concentrations of calcium and sodium were determined using an external calibration (standard curve). The method is validated and accredited, and is based on method NMKL 186, 2007.

Certified reference material for each nutrient analysis was selected based on similarity in concentration and matrix to sample material analyzed. The trueness of each specific method has been tested by analyzing certified reference materials and by participation in proficiency tests. All values of certified reference materials were within the accepted area of analysis.

### Presentation of data

The analytic value given for each seafood product consisted of one composite sample, comprising three different batches. When several products from the same year were present within the similar seafood category (e.g. fish cakes and fish pudding), the mean ± SD value was reported.

We selected one product of fatty fish, mackerel in tomato sauce, and two products of lean fish, fish cakes/burgers (the lean fish product containing the most fish) and fish au gratin (the lean fish product containing the least amount of fish), to estimate how much one portion of the respective products contributed to the recommended intake (RI) for adults (men and women) using the Nordic Nutrition Recommendations (NNR20012) (20). For EPA and DHA, the dietary reference values from the European Food Safety Authority (EFSA) were used (21), as recommendations of EFA and DHA are not present in NNR2012. The following daily RI values for adults were used: EPA+DHA: 250 mg; vitamin D: 10 μg; vitamin B12: 2.0 μg; vitamin A: 800 μg (mean of RI for men (900 μg) and women (700 μg); iodine: 150 μg; selenium: 55 μg (mean of RI for men (60 μg) and women (50 μg); zinc: 8 μg (mean of RI for men (9 μg) and women (7 μg); calcium: 800 mg. Standardized portion sizes from the report ‘Weights, measures and portion sizes for foods’ from the Norwegian Food Safety Authority, University of Oslo, and the Norwegian Directorate of Health were used (22). One portion = 40 g mackerel in tomato sauce, 150 g fish cakes, and 275 g fish au gratin.

## Results

Supplementary Table 1 lists the different seafood products included for analysis, including fish content (%) and fish species used. Most seafood products available for several years had similar fish content; however, some products had an increase in fish content. In the lean seafood products, the most frequently used fish species were greater argentine whole fish (*Argentina silus*), haddock fillet (*Melanogrammus aeglefinus*), Alaska pollock (*Theragra chalcogramma*), saithe fillet (*Pollachius virens*), and cod fillet (*Gadus morhua*). Most of the seafood products of lean fish also combined several types of fish species.

**Table 1a:** Analytic values of total protein, total fat, and fatty acids in 16 different seafood products from 2015 (mean ± SD)^a^

Product^b^	Total protein g/100g	Total fat g/100g	Sum SFA g/100g (%^c^)	Sum MUFA g/100g (%^c^)	Sum PUFA g/100g (%^c^)	Sum n-3 g/100g (%^c^)	Sum n-6 g/100g (%^c^)	EPA mg/100g (%^c^)	DHA mg/100g (%^c^)
‘Berggren’ fish burgers	11.1	4.8	0.5 (9.3)	3.1 (63)	1.4 (27)	0.6 (12)	0.8 (15)	67 (1.3)	150 (3.0)
‘Coop’ fish cakes	11.9	5.3	0.5 (8.4)	3.8 (65)	1.5 (26)	0.6 (9.7)	0.9 (16)	40 (0.7)	120 (2.0)
‘Fiskemannen’ fish cakes	11.1	3.5	0.4 (9.1)	2.9 (64)	1.1 (26)	0.4 (11)	0.7 (15)	41 (1.0)	120 (2.7)
‘Fiskemannen’ fish burgers	12.3	2.5	0.3 (9.9)	2.1 (61)	1.0 (28)	0.4 (12)	0.5 (15)	40 (1.2)	140 (4.0)
‘Godehav’ fish cakes	12.3	3.6	0.6 (11)	3.3 (59)	1.6 (29)	0.6 (12)	1.0 (17)	38 (0.7)	130 (2.3)
‘Godehav’ fish burgers	11.2	3.8	0.6 (10)	3.2 (60)	1.6 (30)	0.6 (11)	1.0 (18)	26 (0.5)	97 (1.8)
‘Lofoten’ fish cakes	11.9	4.4	1.5 (36)	1.8 (43)	0.8 (19)	0.4 (8.7)	0.4 (11)	44 (1.0)	100 (2.5)

**Total fish cakes/burgers (*n* = 7)**	**11.7 ± 0.5**	**4.0 ± 0.9**	**0.6 ± 0.4**	**2.9 ± 0.7**	**1.3 ± 0.30**	**0.5 ± 0.1**	**0.8 ± 0.2**	**42 ± 12**	**120 ± 18**

‘X-tra’ fish au gratin	7.3	4.6	0.5 (12)	2.5 (57)	1.3 (31)	0.5 (11)	0.9 (20)	17 (0.4)	63 (1.5)
‘Enghav’ fish au gratin	7.2	4.8	0.6 (12)	2.6 (57)	1.4 (30)	0.5 (10)	0.9 (20)	20 (0.4)	61 (1.3)
‘Findus’ ‘Familiens’ fish au gratin	8.0	4.7	0.6 (13)	2.7 (56)	1.5 (31)	0.4 (9.2)	1.0 (21)	28 (0.6)	50 (1.0)
‘First Price’ fish au gratin	7.5	4.4	0.5 (12)	2.5 (57)	1.3 (30)	0.5 (11)	0.9 (20)	27 (0.6)	80 (1.8)
‘ICA’ fish au gratin	7.0	5.1	0.5 (12)	2.5 (57)	1.3 (30)	0.4 (10)	0.9 (20)	15 (0.3)	46 (1.0)

**Total fish au gratin (*n* = 5)**	**7.4 ± 0.4**	**4.7 ± 0.3**	**0.6 ± 0.04**	**2.6 ± 0.1**	**1.4 ± 0.1**	**0.5 ± 0.02**	**0.9 ± 0.1**	**21 ± 5.9**	**60 ± 13**

‘Findus’ fish fingers	11.1	7.9	0.6 (9.8)	4.6 (63)	2.0 (28)	0.7 (9.1)	1.4 (19)	54 (0.7)	47 (1.2)
‘Findus’ panned cod fillet	14.3	<1.0^d^	0.1 (21)	0.1 (15)	0.3 (64)	0.2 (35)	0.2 (29)	50 (9.6)	110 (22)

**Total fish fingers (*n* = 2)**	**12.7 ± 2.3**	**4.5 ± 4.9**	**0.4 ± 0.4**	**2.3 ± 3.2**	**1.2 ± 1.2**	**0.4 ± 0.3**	**0.8 ± 0.9**	**52 ± 2.8**	**100 ± 18**

‘First Price’ panned saithe fillet	14.3	7.1	0.6 (8.5)	4.9 (70)	1.5 (22)	0.5 (7.0)	1.0 (15)	60 (0.8)	200 (2.8)
‘Lerøy’ breaded saithe fillet	14.8	8.0	0.7 (9.0)	4.9 (60)	2.4 (30)	0.9 (11)	1.6 (19)	74 (0.9)	200 (2.4)

**Total saithe products (*n* = 2)**	**14.6 ± 0.4**	**7.6 ± 0.64**	**0.7 ± 0.1**	**4.9 ± 0.1**	**2.0 ± 0.6**	**0.7 ± 0.3**	**1.3 ± 0.4**	**67 ± 9.9**	**200 ± 2.1**

a Mean ± SD values are only calculated when several products are compiled.

b The analytic value for each seafood product consists of one composite sample, comprising three different batches.

c Values given in percent of total fatty acids.

d Value below LOQ of 1.0 g/100g.

Tables 1a, 1b, 2a, 2b, 3a, and 3b describe the analytic values for macro- and micronutrients in the different seafood products from 2015, 2017, and 2018, respectively. As seen from Tables 1a, 2a, and 3a, the contents of protein, total fat, and fatty acids were relatively stable within the different categories of seafood products, apart from seafood as spread where large variations were seen within the same product category. The variation of micronutrients, and especially vitamin A, vitamin B12, iodine, and selenium, had large variations both within and between product categories, and also between years.

**Table 1b:** Analytic values of selected vitamins and minerals in 16 different seafood products from 2015 (mean ± SD)^a^

Product ^b^	Vitamin D3 μg/100g	Vitamin A1 μg/100g	Vitamin B12 μg/100g	Iodine μg/100g	Selenium μg/100g	Calcium mg/100g	Zinc mg/100g	Iron mg/100g	Sodium mg/100g
‘Berggren’ fish burgers	<1.0^c^	45	0.8	21	13	39	0.3	0.3	690
‘Coop’ fish cakes	<1.0^c^	9.0	0.9	58	14	28	0.3	0.2	830
‘Fiskemannen’ fish cakes	<1.0^c^	2.4	1.2	33	13	20	0.2	0.2	530
‘Fiskemannen’ fish burgers	<1.0^c^	3.7	1.1	22	15	24	0.2	0.2	430
‘Godehav’ fish cakes	<1.0^c^	5.0	0.5	10	13	53	0.3	0.2	640
‘Godehav’ fish burgers	<1.0^c^	4.7	0.4	11	13	63	0.3	0.1	620
‘Lofoten’ fish cakes	<1.0^c^	20	1.4	110	16	35	0.3	0.1	430

**Total fish cakes/burgers (*n* = 7)**	**<1.0^c^**	**13 ± 15**	**0.9 ± 0.4**	**38 ± 36**	**14 ± 1.2**	**37 ± 16**	**0.3 ± 0.1**	**0.2 ± 0.1**	**600 ± 140**

‘X-tra’ fish au gratin	<1.0^c^	50	0.7	50	8.4	47	0.3	0.2	300
‘Enghav’ fish au gratin	<1.0^c^	45	1.0	21	8.1	43	0.3	0.2	330
‘Findus’ ‘Familiens’ fish au gratin	<1.0^c^	17	0.5	31	17	40	0.6	0.5	310
‘First Price’ fish au gratin	<1.0^c^	50	1.0	40	8.4	40	0.3	0.2	290
‘ICA’ fish au gratin	<1.0^c^	37	0.7	36	7.2	45	0.3	0.3	310

**Total fish au gratin (*n* = 5)**	**<1.0^c^**	**40 ± 14**	**0.8 ± 0.2**	**36 ± 11**	**9.8 ± 4.0**	**43 ± 3.1**	**0.4 ± 0.1**	**0.3 ± 0.1**	**310 ± 15**

‘Findus’ fish fingers	<1.0^c^	7.0	0.8	100	11	20	0.3	0.4	240
‘Findus’ panned cod fillet	<1.0^c^	0.8	0.9	61	14	16	0.4	0.4	150

**Total fish fingers (*n* = 2)**	**<1.0^c^**	**3.9 ± 4.4**	**0.8 ± 0.1**	**81 ± 28**	**13 ± 2.1**	**18 ± 2.8**	**0.4 ± 0.1**	**0.4 ± 0.0**	**200 ± 64**

‘First Price’ panned saithe fillet	<1.0^c^	0.8	1.9	74	15	11	0.5	0.5	180
‘Lerøy’ breaded saithe fillet	<1.0^c^	2.0	2.2	92	23	21	0.5	0.4	270

**Total saithe products (*n* = 2)**	**<1.0^c^**	**1.4 ± 0.9**	**2.1 ± 0.21**	**83 ± 13**	**19 ± 5.7**	**16 ± 7.1**	**0.5 ± 0.0**	**0.5 ± 0.1**	**230 ± 64**

a Mean ± SD values are only calculated when several products are compiled.

b The analytic value for each seafood product consists of one composite sample, comprising three different batches.

c Value below LOQ of 1.0 μg/100g.

**Table 2a:** Analytic values of total protein, total fat, and fatty acids in 35 different seafood products from 2017 (mean ± SD)^a^

Product^b^	Total protein g/100g	Total fat g/100g	Sum SFA g/100g (%^c^)	Sum MUFA g/100g (%^c^)	Sum PUFA g/100g (%^c^)	Sum n-3 g/100g (%^c^)	Sum n-6 g/100g (%^c^)	EPA mg/100g (%^c^)	DHA mg/100g (%^c^)
‘Berggren’ fish burgers	9.3	5.5	0.5 (9.4)	3.1 (60)	1.6 (30)	0.6 (12)	0.9 (18)	62 (1.2)	140 (2.7)
‘Coop’ fish cakes	10.7	6.4	0.4 (8.6)	3.1 (61)	1.4 (29)	0.5 (10)	0.9 (18)	29 (0.6)	95 (1.9)
‘First Price’ fish cakes	9.1	3.6	0.2 (9.2)	1.4 (60)	0.7 (29)	0.3 (12)	0.4 (18)	27 (1.1)	69 (2.9)
‘Fiskemannen’ fish cakes	10.1	4.5	0.3 (9.8)	1.9 (60)	0.9 (29)	0.4 (12)	0.5 (17)	31 (1.0)	98 (3.2)
‘Fiskemannen’ fish burgers	12.9	3.8	0.4 (11)	2.0 (58)	1.0 (30)	0.5 (13)	0.6 (16)	48 (1.4)	156 (4.5)
‘Godehav’ fish cakes	13.3	9.6	0.7 (8.5)	5.4 (63)	2.4 (28)	0.8 (9.3)	1.6 (18)	27 (0.3)	98 (1.1)
‘Godehav’ fish burgers	14.7	5.9	0.6 (11)	3.1 (58)	1.6 (31)	0.7 (13)	1.0 (18)	68 (1.3)	158 (3.0)
‘Lofoten’ fish cakes	11.3	5.4	1.2 (34)	1.6 (44)	0.7 (20)	0.3 (7.9)	0.4 (12)	33 (0.9)	67 (1.9)
‘X-tra’ fish cakes	9.0	7.1	0.5 (9.0)	3.2 (61)	1.5 (28)	0.5 (9.8)	1.0 (19)	27 (0.5)	78 (1.5)

**Total fish cakes/burgers (*n* = 9)**	**11.2 ± 2.1**	**5.8 ± 1.8**	**0.5 ± 0.3**	**2.7 ± 1.2**	**1.3 ± 0.5**	**0.5 ± 0.2**	**0.8 ± 0.4**	**39 ± 16**	**107 ± 36**

‘X-tra’ fish au gratin	6.1	4.8	0.6 (12)	2.8 (57)	1.5 (30)	0.5 (9.4)	1.0 (20)	19 (0.4)	63 (1.3)
‘Findus’ ‘Familiens’ fish au gratin	7.8	5.3	0.6 (12)	2.9 (56)	1.6 (31)	0.4 (8.6)	1.1 (22)	22 (0.4)	48 (0.9)
‘Findus’ ‘God Gammeldags’ fish au gratin	8.5	4.9	0.6 (13)	2.9 (57)	1.5 (30)	0.4 (8.4)	1.1 (21)	18 (0.4)	53 (1.0)
‘First Price’ fish au gratin	6.7	4.5	0.5 (12)	2.4 (57)	1.3 (30)	0.4 (9.6)	0.9 (20)	17 (0.4)	66 (1.6)

**Total fish au gratin (*n* = 4)**	**7.3 ± 1.1**	**4.0 ± 0.3**	**0.6 ± 0.1**	**2.7 ± 0.2**	**1.5 ± 0.1**	**0.4 ± 0.02**	**1.0 ± 0.1**	**19.0 ± 2.2**	**58 ± 8.4**

‘Coop’ fish fingers	10.3	6.8	0.9 (11)	2.2 (28)	4.5 (59)	0.2 (2.2)	4.4 (57)	37 (0.5)	113 (1.5)
‘Findus’ fish fingers	9.4	8.3	0.7 (8.4)	5.5 (62)	2.5 (29)	0.8 (8.5)	1.8 (20)	42 (0.5)	85 (1.0)
‘First Price’ fish fingers	10.1	8.3	0.7 (8.4)	5.1 (62)	2.4 (29)	0.7 (8.9)	1.6 (20)	37 (0.4)	85 (1.0)
‘Lerøy’ fish fingers	11.4	7.6	0.6 (8.1)	4.5 (62)	2.1 (29)	0.7 (9.8)	1.4 (19)	47 (0.6)	112 (1.5)
‘Findus’ panned cod fillet	12.8	0.7	0.1 (20)	0.1 (12)	0.3 (65)	0.2 (35)	0.2 (30)	48 (9.3)	112 (22.0)

**Total fish fingers (*n* = 5)**	**10.8 ± 1.3**	**6.3 ± 3.2**	**0.6 ± 0.3**	**3.5 ± 2.3**	**2.4 ± 1.5**	**0.5 ± 0.3**	**1.9 ± 1.5**	**42 ± 5.3**	**101 ± 15**

‘First Price’ panned saithe fillet	13.3	6.9	0.6 (8.4)	5.3 (70)	1.6 (21)	0.5 (6.4)	1.1 (14)	53 (0.7)	208 (2.7)

**Total saithe products (*n* = 1)**	**NA**	**NA**	**NA**	**NA**	**NA**	**NA**	**NA**	**NA**	**NA**

‘First Price’ fish pudding	6.9	6.0	0.7 (12)	3.5 (59)	1.7 (28)	0.6 (10)	1.1 (18)	37 (0.6)	77 (1.3)
‘Fiskemannen’ fish pudding	8.0	6.3	0.8 (12)	3.8 (59)	1.8 (27)	0.6 (9.5)	1.2 (18)	28 (0.4)	72 (1.1)
‘Godehav’ fish pudding	11.7	4.2	0.8 (21)	2.1 (53)	1.0 (25)	0.4 (11)	0.6 (14)	38 (0.9)	135 (3.4)

**Total fish pudding (*n* = 3)**	**8.9 ± 2.5**	**5.5 ± 1.1**	**0.8 ± 0.1**	**3.2 ± 0.9**	**1.5 ± 0.4**	**0.6 ± 0.1**	**0.9 ± 0.3**	**34 ± 5.5**	**95 ± 35**

‘Fiskemannen’ smoked salmon	23.0	3.4	1.1 (16)	3.1 (46)	2.2 (33)	1.2 (18)	1.0 (15)	184 (2.7)	445 (6.6)
‘Godehav’ smoked trout	24.0	1.2	1.3 (17)	3.8 (49)	2.5 (32)	1.4 (18)	1.1 (14)	234 (3.0)	627 (8.0)
‘Lerøy’ smoked salmon	20.0	7.8	1.6 (16)	5.1 (50)	3.3 (33)	1.7 (17)	1.5 (15)	325 (3.2)	495 (4.9)
‘Lerøy’ smoked trout	20.0	3.2	1.4 (17)	4.4 (51)	2.7 (31)	1.5 (18)	1.2 (14)	221 (2.6)	620 (7.2)
‘Lofoten’ smoked salmon	24.0	6.8	1.2 (15)	3.8 (46)	2.8 (34)	1.5 (18)	1.3 (16)	244 (3.0)	483 (5.9)
‘Stabburet’ canned hot-smoked salmon	12.4	17.1	2.0 (12)	9.1 (55)	5.0 (30)	1.0 (12)	3.0 (18)	227 (1.4)	624 (2.2)

**Total spread salmon/trout (*n* = 6)**	**20.6 ± 4.4**	**6.6 ± 5.7**	**1.4 ± 0.3**	**4.9 ± 2.2**	**3.1 ± 1.0**	**1.5 ± 0.3**	**1.5 ± 0.8**	**239 ± 47**	**505 ± 102**

‘Coop’ mackerel in tomato sauce	11.6	21.0	4.2 (22)	8.5 (44)	6.1 (31)	5.1 (26)	1.0 (5.2)	1245 (6.4)	2157 (11.1)
‘King Oscar’ mackerel in tomato sauce	8.0	14.8	2.9 (22)	5.9 (44)	4.2 (31)	3.6 (27)	0.6 (4.1)	937 (7.0)	1533 (11.4)
‘Stabburet’ mackerel in tomato sauce	12.4	21.0	3.8 (20)	8.9 (47)	5.8 (31)	4.8 (25)	1.1 (5.5)	1234 (6.4)	1918 (10.0)

**Total spread mackerel in tomato sauce (*n* = 3)**	**10.7 ± 2.3**	**18.9 ± 3.6**	**3.6 ± 0.6**	**7.8 ± 1.6**	**5.4 ± 1.0**	**4.5 ± 0.7**	**0.9 ± 0.3**	**1139 ± 175**	**1869 ± 315**

‘First Price’ caviar	7.8	38.0	2.7 (7.6)	22.2 (62)	10.3 (29)	3.6 (10)	6.7 (19)	149 (0.4)	270 (0.8)
‘Kavli’ caviar	11.9	15.4	2.3 (8.4)	16.6 (63)	7.4 (27)	2.6 (10)	4.7 (18)	229 (0.9)	394 (1.5)
‘Mills’ caviar	8.8	38.0	3.0 (7.7)	23.8 (61)	11.6 (30)	4.2 (11)	7.4 (19)	185 (0.5)	310 (0.8)
‘Rema 1000’ caviar	6.4	39.0	2.7 (7.5)	22.7 (63)	10.2 (28)	3.4 (9.5)	6.8 (19)	148 (0.4)	257 (0.7)

**Total spread caviar (*n* = 4)**	**8.7 ± 2.3**	**32.6 ± 11.5**	**2.7 ± 0.3**	**21.4 ± 3.1**	**9.9 ± 1.8**	**3.5 ± 0.6**	**6.4 ± 1.2**	**178 ± 38**	**308 ± 62**

a Mean ± SD values are only calculated when several products are compiled.

b The analytic value for each seafood product consists of one composite sample, comprising three different batches.

c Values given in percent of total fatty acids. NA: Not applicable.

**Table 2b:** Analytic values of selected vitamins and minerals in 35 different seafood products from 2017 (mean ± SD)^a^

Product^b^	Vitamin D3 μg/100g	Iodine μg/100g	Selenium μg/100g	Calcium mg/100g	Zinc mg/100g	Iron mg/100g	Sodium mg/100g
‘Berggren’ fish burgers	1.0	19	12	40	0.3	0.2	740
‘Coop’ fish cakes	2.0	23	15	29	0.3	0.2	600
‘First Price’ fish cakes	2.0	120	14	23	0.2	0.2	510
‘Fiskemannen’ fish cakes	3.0	68	15	25	0.2	0.1	480
‘Fiskemannen’ fish burgers	5.0	54	18	67	0.3	0.2	540
‘Godehav’ fish cakes	<1.0^c^	7.5	19	12	0.3	0.1	570
‘Godehav’ fish burgers	4.0	110	23	20	0.4	0.2	380
‘Lofoten’ fish cakes	1.0	76	18	42	0.3	0.2	580
‘X-tra’ fish cakes	<1.0^c^	19	12	34	0.2	0.2	650

**Total fish cakes/burgers (*n* = 9)**	**2.2 ± 1.5**	**55 ± 41**	**16 ± 4**	**32 ± 16**	**0.3 ± 0.1**	**0.2 ± 0.03**	**560 ± 100**

‘X-tra’ fish au gratin	2.0	24	7.9	45	0.3	0.3	340
‘Findus’ ‘Familiens’ fish au gratin	<1.0^c^	18	7.4	48	0.6	0.5	340
‘Findus’ ‘God Gammeldags’ fish au gratin	<1.0^c^	45	9.0	45	0.6	0.5	320
‘First Price’ fish au gratin	2.0	35	9.8	42	0.3	0.3	320

**Total fish au gratain (*n* = 4)**	**1.5 ± <1.0^c^**	**31 ± 12**	**9 ± 1**	**45 ± 2.4**	**0.4 ± 0.1**	**0.4 ± 0.1**	**330 ± 12**

‘Coop’ fish fingers	<1.0^c^	46	13	20	0.5	0.4	140
‘Findus’ fish fingers	<1.0^c^	88	13	17	0.4	0.3	320
‘First Price’ fish fingers	<1.0^c^	31	13	14	0.4	0.5	370
‘Lerøy’ fish fingers	<1.0^c^	120	14	18	0.5	0.4	360
‘Findus’ panned cod fillet	<1.0^c^	32	13	17	0.4	0.4	230

**Total fish fingers (*n* = 5)**	**<1.0^c^**	**63 ± 39**	**13 ± 0.4**	**17 ± 2.2**	**0.4 ± 0.1**	**0.4 ± 0.1**	**280 ± 98**

‘First Price’ panned saithe fillet	1.0	57	16	11	0.6	0.5	180

**Total saithe products (*n* = 1)**	**NA**	**NA**	**NA**	**NA**	**NA**	**NA**	**NA**

‘First Price’ fish pudding	<1.0^c^	42	8.4	84	0.2	0.2	830
‘Fiskemannen’ fish pudding	<1.0^c^	72	10	89	0.2	0.2	910
‘Godehav’ fish pudding	<1.0^c^	15	14	61	0.3	0.2	450

**Total fish pudding (*n* = 3)**	**<1.0^c^**	**43 ± 29**	**11 ± 3**	**78 ± 15**	**0.3 ± 0.1**	**0.2 ± 0.01**	**730 ± 250**

‘Fiskemannen’ smoked salmon	2.0	5.7	19	7.8	0.4	0.3	1100
‘Godehav’ smoked trout	8.0	7.6	22	12	0.4	0.3	1200
‘Lerøy’ smoked salmon	2.0	3.1	11	5.6	0.3	0.2	1200
‘Lerøy’ smoked trout	3.0	4.7	12	7.6	0.3	0.2	1200
‘Lofoten’ smoked salmon	3.0	5.2	11	9.1	0.4	0.3	1500
‘Stabburet’ canned hot-smoked salmon	5.0	2.6	10	9.8	0.3	0.2	590

**Total spread- salmon/trout (*n* = 6)**	**3.8 ± 2.3**	**5 ± 2**	**14 ± 5**	**8.7 ± 2.2**	**0.4 ± 0.1**	**0.2 ± 0.1**	**1100 ± 300**

‘Coop’ mackerel in tomato sauce	3.0	39	29	17	0.9	0.8	430
‘King Oscar’ mackerel in tomato sauce	3.0	31	19	18	0.5	0.6	500
‘Stabburet’ mackerel in tomato sauce	3.0	56	28	17	0.9	0.8	360

**Total spread – mackerel in tomato sauce (*n* = 3)**	**3.0 ± 0.0**	**42 ± 13**	**25 ± 6**	**17 ± 0.6**	**0.8 ± 0.2**	**0.8 ± 0.1**	**430 ± 70**

‘First Price’ caviar	3.0	51	26	4.8	1.6	0.3	2400
‘Kavli’ caviar	2.0	79	38	6.2	2.6	0.4	3200
‘Mills’ caviar	3.0	63	35	4.0	2.2	0.3	2400
‘Rema 1000’ caviar	3.0	52	23	5.2	1.6	0.3	2500

**Total spread – caviar (*n* = 4)**	**2.8 ± 0.5**	**61 ± 13**	**31 ± 7**	**5.1 ± 0.9**	**2.0 ± 0.5**	**0.3 ± 0.1**	**2600 ± 390**

a Mean ± SD values are only calculated when several products are compiled.

b The analytic value for each seafood product consists of one composite sample, comprising three different batches.

c Value below LOQ of 1.0 μg/100g.

**Table 3a:** Analytic values of macronutrients and fatty acids in 35 different seafood products from 2018 (mean ±SD)^a^

Product^b^	Total protein g/100g	Total fat g/100g	Sum SFA g/100g (%^c^)	Sum MUFA g/100g (%^c^)	Sum PUFA g/100g (%^c^)	Sum n-3 g/100g (%^c^)	Sum n-6 g/100g (%^c^)	EPA mg/100g (%^c^)	DHA mg/100g (%^c^)
‘Best pris’ fish cakes	10.8	6.6	0.6 (9.7)	3.9 (62)	1.8 (28)	0.6 (10)	1.1 (18)	38 (0.6)	125 (2.0)
‘Coop’ fish cakes	11.8	7.0	0.6 (8.8)	4.4 (63)	1.9 (27)	0.7 (9.6)	1.2 (18)	48 (0.7)	144 (2.1)
‘First Price’ fish cakes	9.8	3.9	0.4 (9.1)	2.4 (62)	1.1 (28)	0.5 (12)	0.7 (17)	42 (1.1)	120 (3.1)
‘Fiskemannen’ fish cakes	13.4	5.8	0.4 (10)	2.3 (60)	1.1 (29)	0.5 (13)	0.6 (16)	60 (1.5)	153 (4.0)
‘Fiskemannen’ fish burgers	12.9	4.2	0.4 (10)	2.5 (59)	1.2 (30)	0.5 (13)	0.7 (17)	50 (1.2)	168 (4.0)
‘Lofoten’ fish burgers 86%	15.3	5.5	1.5 (28)	2.6 (49)	1.1 (21)	0.5 (9.0)	0.7 (12)	56 (1.1)	163 (3.1)
‘Lofoten’ fish cakes	11.6	5.6	1.8 (32)	2.6 (48)	1.0 (18)	0.4 (7.0)	0.6 (11)	45 (0.8)	97 (1.8)
‘X-tra’ fish cakes	11.6	7.1	0.8 (12)	4.1 (63)	1.6 (25)	0.5 (8.2)	1.1 (17)	40 (0.6)	121 (1.9)
‘Rema 1000’ fish burgers XXL	10.0	6.0	0.5 (9.0)	3.5 (62)	1.6 (28)	0.6 (9.9)	1.0 (18)	35 (0.6)	96 (1.7)

**Total fish cakes/burgers (*n* = 9)**	**11.9 ± 1.7**	**5.7 ± 1.1**	**0.8 ± 0.5**	**3.2 ± 0.8**	**1.4 ± 0.3**	**0.5 ± 0.1**	**0.9 ± 0.3**	**46 ± 8.3**	**132 ± 27**

‘X-tra’ fish au gratin	8.8	4.9	0.6 (12)	3.0 (59)	1.5 (29)	0.5 (11)	0.9 (18)	24 (0.5)	102 (2.0)
‘Findus’ ‘Familiens’ fish au gratin	8.6	5.0	0.6 (12)	2.7 (58)	1.4 (30)	0.4 (8.9)	1.0 (21)	26 (0.6)	45 (1.0)
‘Findus’ ‘God Gammeldags’ fish au gratin	8.4	5.8	0.7 (12)	3.3 (58)	1.7 (30)	0.5 (9.3)	1.2 (20)	17 (0.3)	59 (1.0)
‘First Price’ fish au gratin	8.8	4.2	0.5 (12)	2.4 (58)	1.2 (30)	0.4 (10)	0.8 (20)	21 (0.5)	72 )1.8)

**Total fish au gratin (*n* = 4)**	**8.7 ± 0.2**	**5.0 ± 0.7**	**0.6 ± 0.1**	**2.8 ± 0.4**	**1.5 ± 0.2**	**0.5 ± 0.1**	**1.0 ± 0.1**	**22 ± 3.9**	**70 ± 24**

‘Findus’ fish fingers	11.5	8.9	0.7 (8.3)	5.4 (63)	2.4 (28)	0.7 (8.5)	1.7 (20)	54 (0.6)	109 (1.3)
‘First Price’ fish fingers	10.9	8.9	0.7 (8.1)	4.9 (61)	2.5 (31)	0.8 (10)	1.7 (20)	63 (0.8)	95 (1.2)
‘Lerøy’ fish fingers	12.7	7.7	0.9 (11)	2.4 (32)	4.2 (56)	0.2 (2.7)	4.0 (54)	51 (0.7)	120 (1.6)
‘X-tra’ fish fingers	11.6	7.9	0.7 (8.5)	4.8 (63)	2.1 (28)	0.7 (8.9)	1.4 (19)	43 (0.6)	110 (1.5)
‘Findus’ panned cod fillet	14.2	0.8	0.1 (20)	0.1 (12)	0.4 (67)	0.2 (35)	0.2 (32)	58 (9.8)	133 (22)

**Total fish fingers (*n* = 5)**	**12.2 ± 1.3**	**6.8 ± 3.4**	**0.6 ± 0.3**	**3.5 ± 2.3**	**2.3 ± 1.3**	**0.5 ± 0.3**	**1.8 ± 1.4**	**54 ± 7.5**	**113 ± 14**

‘First Price’ panned saithe fillet	14.1	6.8	0.5 (8.3)	4.6 (72)	1.2 (19)	0.4 (6.4)	0.8 (12)	60 (0.9)	209 (3.3)

**Total saithe products (*n* = 1)**	**NA**	**NA**	**NA**	**NA**	**NA**	**NA**	**NA**	**NA**	**NA**

‘First Price’ fish pudding	7.3	6.2	0.7 (12)	3.5 (60)	1.6 (28)	0.6 (9.8)	1.1 (18)	27 (0.5)	57 (1.0)
‘Fiskemannen’ fish pudding	8.4	6.2	0.7 (12)	3.7 (60)	1.7 (28)	0.6 (9.8)	1.1 (18)	37 (0.6)	71 (1.1)
‘Best pris’ fish pudding	10.5	3.3	0.6 (18)	1.8 (56)	0.8 (25)	0.3 (11)	0.5 (14)	37 (1.2)	123 (3.8)

**Total fish pudding (*n* = 3)**	**8.7 ± 1.6**	**5.2 ± 1.7**	**0.7 ± 0.1**	**3.0 ± 1.0**	**1.4 ± 0.5**	**0.5 ± 0.1**	**0.9 ± 0.4**	**34 ± 5.8**	**84 ± 35**

‘Fiskemannen’ smoked salmon	22.0	9.3	1.3 (15)	4.5 (52)	2.8 (32)	1.4 (17)	1.4 (16)	239 (2.8)	496 (5.7)
‘Godehav’ smoked trout	23.0	16.0	2.5 (17)	7.3 (51)	4.6 (32)	2.5 (17)	2.0 (14)	465 (3.2)	794 (5.5)
‘Lofoten’ smoked salmon	24.0	10.7	1.6 (15)	5.5 (52)	3.4 (32)	1.7 (16)	1.7 (16)	295 (2.8)	521 (4.9)
‘Stabburet’ canned hot-smoked salmon	15.9	17.2	2.0 (12)	9.7 (57)	5.3 (31)	2.2 (13)	3.1 (18)	240 (1.4)	392 (2.3)

**Total spread – salmon/trout (*n* = 4)**	**21.2 ± 3.6**	**13.3 ± 3.9**	**1.9 ± 0.5**	**6.7 ± 2.3**	**4.0 ± 1.1**	**1.9 ± 0.5**	**2.0 ± 0.8**	**310 ± 107**	**551 ± 172**

‘Coop’ mackerel in tomato sauce	11.9	20.0	3.8 (20)	8.4 (44)	6.5 (34)	5.5 (29)	0.9 (4.9)	1313 (6.9)	2162 (11)
‘King Oscar’ mackerel in tomato sauce	10.6	18.2	2.8 (18)	7.8 (49)	4.9 (31)	3.7 (23)	1.2 (7.6)	841 (5.4)	1336 (8.5)
‘Stabburet’ mackerel in tomato sauce	11.9	19.0	3.2 (18)	8.4 (48)	5.5 (32)	4.2 (24)	1.2 (7.0)	1017 (5.9)	1529 (8.9)

**Total spread – mackerel in tomato sauce (*n* = 3)**	**11.4 ± 0.8**	**19.1 ± 0.9**	**3.3 ± 0.5**	**8.2 ± 0.4**	**5.6 ± 0.8**	**4.4 ± 0.9**	**1.1 ± 0.1**	**1057 ± 239**	**1676 ± 432**

‘First Price’ caviar	7.3	37.0	2.8 (7.5)	23.1 (62)	10.9 (30)	4.0 (11)	7.0 (19)	127 (0.3)	314 (0.8)
‘Kavli’ caviar	12.1	26.0	2.0 (7.9)	15.7 (61)	7.9 (31)	3.1 (12)	4.8 (19)	227 (0.9)	406 (1.6)
‘Mills’ caviar	9.1	38.0	2.8 (7.5)	22.8 (62)	10.9 (30)	3.9 (11)	7.0 (19)	174 (0.5)	328 (0.9)
‘Rema 1000’ caviar	8.5	37.0	2.7 (7.5)	22.2 (63)	10.4 (29)	3.6 (11)	6.8 (19)	144 (0.4)	245 (0.7)

**Total spread – caviar (*n* = 4)**	**9.3 ± 2.0**	**34.5 ± 3.7**	**2.6 ± 0.4**	**21.0 ± 3.5**	**10.0 ± 1.5**	**3.6 ± 0.4**	**6.4 ± 1.1**	**168 ± 44**	**323 ± 66**

a Mean ± SD values are only calculated when several products are compiled.

b The analytic value for each seafood product consists of one composite sample, comprising three different batches where four different packages were included in each batch.

c Values given in percent of total fatty acids.

**Table 3b:** Analytic values of selected vitamins and minerals in 35 different seafood products from 2018 (mean ± SD)^a^

Product^b^	Vitamin D3 μg/100g	Iodine μg/100g	Selenium μg/100g	Calcium mg/100g	Sodium mg/100g
‘Best pris’ fish cakes	<1.0^c^	13	13	64	420
‘Coop’ fish cakes	2.0	33	17	51	730
‘First Price’ fish cakes	5.0	37	15	16	590
‘Fiskemannen’ fish cakes	6.0	60	22	59	710
‘Fiskemannen’ fish burgers	2.0	35	18	17	570
‘Lofoten’ fish burgers 86%	1.0	59	22	22	460
‘Lofoten’ fish cakes	<1.0^c^	95	17	65	680
‘X-tra’ fish cakes	<1.0^c^	140	20	53	870
‘Rema 1000’ fish burgers XXL	1.0	39	13	56	460

**Total fish cakes/burgers (*n* = 9)**	**2.2 ± 1.9**	**57 ± 39**	**17 ± 3**	**45 ± 20**	**610 ± 150**

‘X-tra’ fish au gratin	1.0	34	11	79	320
‘Findus’ ‘Familiens’ fish au gratin	<1.0^c^	23	7.3	43	360
‘Findus’ ‘God Gammeldags’ fish au gratin	<1.0^c^	49	8.7	42	270
‘First Price’ fish au gratin	2.0	39	9.2	70	300

**Total fish au gratin (*n* = 4)**	**1.3 ± <1.0^c^**	**36 ± 11**	**9 ± 2**	**59 ± 19**	**310 ± 38**

‘Findus’ fish fingers	<1.0^c^	52	14	16	250
‘First Price’ fish fingers	<1.0^c^	37	12	18	260
‘Lerøy’ fish fingers	<1.0^c^	53	13	14	360
‘X-tra’ fish fingers	<1.0^c^	57	12	42	270
‘Findus’ panned cod fillet	<1.0^c^	55	15	19	170

**Total fish fingers (*n* = 5)**	**<1.0^c^**	**51 ± 8**	**13 ± 1**	**22 ± 11**	**260 ± 68**

‘First Price’ panned saithe fillet	1.0	85	20	10	170

**Total saithe products (*n* = 1)**	**NA**	**NA**	**NA**	**NA**	**NA**

‘First Price’ fish pudding	<1.0^c^	44	10	170	1100
‘Fiskemannen’ fish pudding	1.0	49	11	120	980
‘Best pris’ fish pudding	<1.0^c^	14	12	80	510

**Total fish pudding (*n* = 3)**	**<1.0^c^**	**36 ± 19**	**11 ± 1**	**123 ± 45**	**860 ± 310**

‘Fiskemannen’ smoked salmon	6.0	4.9	19	9.4	1200
‘Godehav’ smoked trout	4.0	12	19	10	890
‘Lofoten’ smoked salmon	5.0	6.0	15	7.8	1300
‘Stabburet’ canned hot-smoked salmon	4.0	4.0	15	12	580

**Total spread – salmon/trout (*n* = 4)**	**4.8 ± 1.0**	**7 ± 4**	**17 ± 2**	**9.8 ± 1.7**	**990 ± 330**

‘Coop’ mackerel in tomato sauce	2.0	10	28	35	510
‘King Oscar’ mackerel in tomato sauce	3.0	7.6	24	19	470
‘Stabburet’ mackerel in tomato sauce	2.0	10	23	24	430

**Total spread – mackerel in tomato sauce (*n* = 3)**	**2.3 ± 0.6**	**9 ± 1**	**25 ± 3**	**26 ± 8.2**	**470 ± 40**

‘First Price’ caviar	6.0	41	33	5.1	1900
‘Kavli’ caviar	1.0	100	38	6	2700
‘Mills’ caviar	2.0	63	31	4.2	2100
‘Rema 1000’ caviar	5.0	52	NA	4.7	2200

**Total spread – caviar (*n* = 4)**	**3.0 ± 2.6**	**68 ± 30**	**34 ± 4**	**5.1 ± 0.8**	**2200 ± 340**

a Mean ± SD values are only calculated when several products are compiled.

b The analytic value for each seafood product consists of one composite sample, comprising three different batches.

c Value below LOQ of 1.0 μg/100g.

In Table 4, the nutrient content is presented for the different categories of compiled seafood products from 2015, 2017, and 2018. There were small variations in total protein content between the different years in the different categories of compiled seafood products. The total content of fat, SFA, MUFA, PUFA, n-3, and n-6 of lean seafood products had small variations between years. In contrast, the total fat content of different spreads showed large variations between 2017 and 2018, of which all products had a higher total fat content in 2018.

**Table 4:** Mean ± SD nutrient content of the different categories of compiled seafood products within the same category from 2015, 2017, and 2018^a^

Nutrients	Year	Fish cakes/burgers	Fish au gratain	Fish fingers	Fish pudding	Spread salmon/trout	Spread mackerel in tomato sauce	Spread caviar
Total protein g/100g	2015	11.7 ± 0.5	7.4 ± 0.4	12.7 ± 2.3	NA^b^	NA^b^	NA^b^	NA^b^
	2017	11.2 ± 2.1	7.3 ± 1.1	10.8 ± 1.3	8.9 ± 2.5	20.6 ± 4.4	10.7 ± 2.3	8.7 ± 2.3
	2018	11.9 ± 1.7	8.7 ± 0.2	12.2 ± 1.3	8.7 ± 1.6	21.2 ± 3.6	11.4 ± 0.8	9.3 ± 2.0

Total fat g/100g	2015	4.0 ± 0.9	4.7 ± 0.3	4.5 ± 4.9	NA^b^	NA^b^	NA^b^	NA^b^
	2017	5.8 ± 1.8	4.0 ± 0.3	6.3 ± 3.2	5.5 ± 1.1	6.6 ± 5.7	10.7 ± 2.3	8.7 ± 2.3
	2018	5.7 ± 1.1	5.0 ± 0.7	6.8 ± 3.4	5.2 ± 1.7	13.3 ± 3.9	19.1 ± 0.9	34.5 ± 3.7

Sum SFA g/100g	2015	0.6 ± 0.4	0.6 ± 0.4	0.4 ± 0.4	NA^b^	NA^b^	NA^b^	NA^b^
	2017	0.5 ± 0.3	0.6 ± 0.1	0.6 ± 0.3	0.8 ± 0.1	1.4 ± 0.3	3.6 ± 0.6	2.7 ± 0.3
	2018	0.8 ± 0.5	0.6 ± 0.1	0.6 ± 0.3	0.7 ± 0.1	1.9 ± 0.5	3.3 ± 0.5	2.6 ± 0.4

Sum MUFA g/100g	2015	2.9 ± 0.7	2.6 ± 0.1	2.3 ± 3.2	NA^b^	NA^b^	NA^b^	NA^b^
	2017	2.7 ± 1.2	2.7 ± 0.2	3.5 ± 2.3	3.2 ± 0.9	4.9 ± 2.2	7.8 ± 1.6	21.4 ± 3.1
	2018	3.2 ± 0.8	2.8 ± 0.4	3.5 ± 2.3	3.0 ± 1.0	6.7 ± 2.3	19.1 ± 0.9	34.5 ± 3.7

Sum PUFA g/100g	2015	1.3 ± 0.3	1.4 ± 0.1	1.2 ± 1.2	NA^b^	NA^b^	NA^b^	NA^b^
	2017	1.3 ± 0.5	1.5 ± 0.1	2.4 ± 1.5	1.5 ± 0.4	3.1 ± 1.0	5.4 ± 1.0	9.9 ± 1.8
	2018	1.4 ± 0.3	1.5 ± 0.2	2.3 ± 1.3	1.4 ± 0.5	4.0 ± 1.1	5.6 ± 0.8	10.0 ± 1.5

Sum n-3 g/100g	2015	0.5 ± 0.1	0.5 ± 0.02	0.4 ± 0.3	NA^b^	NA^b^	NA^b^	NA^b^
	2017	0.5 ± 0.2	0.4 ± 0.02	0.5 ± 0.3	0.6 ± 0.1	1.5 ± 0.3	4.5 ± 0.7	3.5 ± 0.6
	2018	0.5 ± 0.1	0.5 ± 0.1	0.5 ± 0.3	0.5 ± 0.1	1.9 ± 0.5	4.4 ± 0.9	3.6 ± 0.4

Sum *n*-6 g/100g	2015	0.8 ± 0.2	0.9 ± 0.1	0.8 ± 0.9	NA^b^	NA^b^	NA^b^	NA^b^
	2017	0.8 ± 0.4	1.0 ± 0.1	1.9 ± 1.5	0.9 ± 0.3	1.5 ± 0.8	0.9 ± 0.3	6.4 ± 1.2
	2018	0.9 ± 0.3	1.0 ± 0.1	1.8 ± 1.4	0.9 ± 0.4	2.0 ± 0.8	1.1 ± 0.1	6.4 ± 1.1

EPA mg/100g	2015	42 ± 12	21 ± 5.9	52 ± 2.8	NA^b^	NA^b^	NA^b^	NA^b^
	2017	39 ± 16	19 ± 2.2	42 ± 5.3	34 ± 5.5	239 ± 47	1139 ± 175	178 ± 38
	2018	46 ± 8.3	22 ± 3.9	54 ± 7.5	34 ± 5.8	310 ± 107	1057 ± 239	168 ± 44

DHA mg/100g	2015	120 ± 18	60 ± 13	100 ± 18	NA^b^	NA^b^	NA^b^	NA^b^
	2017	107 ± 36	58 ± 8.4	101 ± 15	95 ± 35	505 ± 102	1869 ± 315	38 ± 62
	2018	132 ± 27	70 ± 24	113 ± 14	84 ± 35	551 ± 172	1676 ± 432	323 ± 66

Vitamin D3 μg/100g	2015	<1.0	<1.0	<1.0	NA^b^	NA^b^	NA^b^	NA^b^
	2017	2.2 ± 1.5	1.5 ± <1.0	<1.0	<1.0	3.8 ± 2.3	3.0 ± 0.0	2.8 ± 0.5
	2018	2.2 ± 1.9	1.3 ± <1.0	<1.0	<1.0	4.8 ± 1.0	2.3 ± 0.6	3.0 ± 2.6

Iodine μg/100g	2015	38 ± 36	36 ± 11	81 ± 28	NA^b^	NA^b^	NA^b^	NA^b^
	2017	55 ± 41	31 ± 12	63 ± 39	43 ± 29	5 ± 2	42 ± 13	61 ± 13
	2018	57 ± 39	36 ± 11	51 ± 8	36 ± 19	7 ± 4	9 ± 1	68 ± 30

Selenium μg/100g	2015	14 ± 1.2	9.8 ± 4.0	13 ± 2.1	NA^b^	NA^b^	NA^b^	NA^b^
	2017	16 ± 4	9 ± 1	13 ± 0.4	11 ± 3	14 ± 5	25 ± 6	31 ± 7
	2018	17 ± 3	9 ± 2	13 ± 1	11 ± 1	17 ± 2	25 ± 3	34 ± 4

Calcium mg/100g	2015	37 ± 16	43 ± 3.1	18 ± 2.8	16 ± 7.1	NA^b^	NA^b^	NA^b^
	2017	32 ± 16	45 ± 2.4	17 ± 2.2	78 ± 15	8.7 ± 2.2	17 ± 0.6	5.1 ± 0.9
	2018	45 ± 20	59 ± 19	22 ± 11	123 ± 45	9.8 ± 1.7	26 ± 8.2	5.1 ± 0.8

Zinc mg/100g	2015	0.3 ± 0.1	0.4 ± 0.1	0.4 ± 0.1	NA^b^	NA^b^	NA^b^	NA^b^
	2017	0.3 ± 0.1	0.4 ± 0.1	0.4 ± 0.1	0.3 ± 0.1	0.4 ± 0.1	0.8 ± 0.2	1.0 ± 0.5
	2018	NA^c^	NA^c^	NA^c^	NA^c^	NA^c^	NA^c^	NA^c^

Iron mg/100g	2015	0.1 ± 0.1	0.3 ± 0.1	0.4 ± 0.0	NA^b^	NA^b^	NA^b^	NA^b^
	2017	0.2 ± 0.03	0.4 ± 0.1	0.4 ± 0.1	0.2 ± 0.01	0.2 ± 0.1	0.8 ± 0.1	0.3 ± 0.1
	2018	NA^c^	NA^c^	NA^c^	NA^c^	NA^c^	NA^c^	NA^c^

Sodium mg/100g	2015	600 ± 140	310 ± 15	200 ± 64	230 ± 64	NA^b^	NA^b^	NA^b^
	2017	560 ± 100	330 ± 12	280 ± 98	730 ± 250	1100 ± 300	430 ± 70	2600 ± 390
	2018	610 ± 150	310 ± 38	260 ± 68	860 ± 310	990 ± 330	470 ± 40	2200 ± 340

a Values given as mean ± SD for compiled seafood products within the same category, each seafood product consists of one composite sample, comprising three different batches.

b Not applicable as these products were not collected in 2015.

c Zinc and iron were not analyzed in 2018. Number of composite samples of: Fish cakes/ burgers: 2015, *n* = 7; 2017 and 2018, *n* = 9. Fish au gratain: 2015, *n* = 5; 2017 and 2018, *n* = 4. Fish fingers: 2015, *n* = 2; 2017 and 2018, *n* = 5. Fish pudding: 2017 and 2018, *n* = 3. Spread, salmon/ trout: 2017, *n* = 6; 2018, *n* = 4. Spread, mackerel in tomato sauce: 2017, *n* = 3; 2018, *n* = 4. Spread, caviar: 2017 and 2018, *n* = 4. Saithe products were not included in the table as there were only two samples in 2015 and one from 2017 and 2018.

**Fig. 1:**
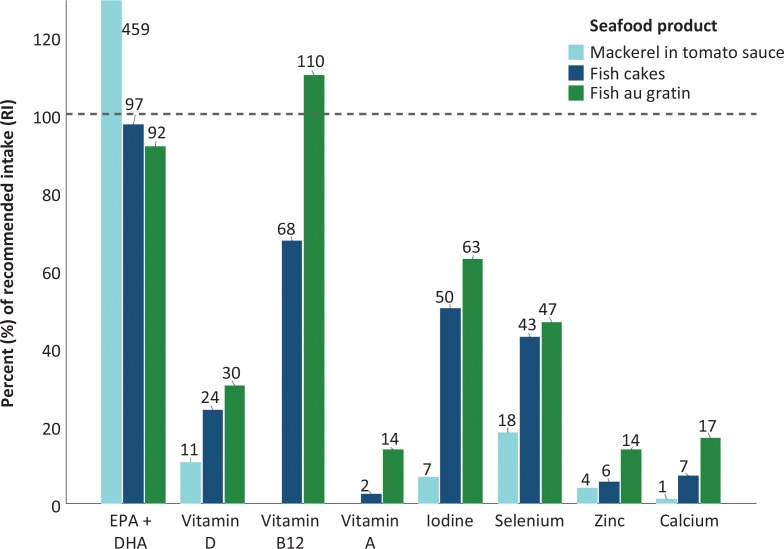
Percent of daily recommended intake (RI) of one portion of mackerel in tomato sauce, fish cakes, and fish au gratin of different micronutrients. Percentages are compared to the daily RI values of adults from NNR 2012 (20), with the exception of EPA+DHA where the Dietary Reference Value (DRV) from EFSA is used (21). One portion = 40 g mackerel in tomato sauce, 150 g fish cakes, and 275 g fish au gratin (22). Values above 120% were compressed in the figure (one value EPA+DHA for mackerel in tomato sauce: 459%). Analyses of vitamin B12 and vitamin A in mackerel in tomato sauce were not available.

Figure 1 illustrates in percent the contribution of one portion of the seafood categories mackerel in tomato sauce, fish cakes, and fish au gratin compared with the daily dietary RI of adults from NNR 2012 and EFSA. As seen in the figure, one portion of mackerel in tomato contributed the most with EPA and DHA (459% of RI). However, also the lean fish products fulfilled the RI of EPA and DHA if one consumed one portion. Generally, the lean fish product had the largest contribution to the RI values for vitamins and minerals.

## Discussion

In this study, we have analyzed an extensive amount of nutrients in a comprehensive selection of seafood products available for Norwegian consumers. This study confirms that seafood products are a good source of several key nutrients, such as EPA and DHA, vitamin D, vitamin B12, iodine, and selenium. However, the nutrient content varies between the different categories of seafood products.

The total fat content of different seafood spreads showed large variations between 2017 and 2018, of which all products had a higher total fat content in 2018. We cannot conclude on whether this was caused by natural variation of the fish used in the products, changes in the nutrient content in other ingredients, or a change in recipe. However, the labeled content and type of fish used were similar between the two years. The seafood spreads were only collected in 2017 and 2018; thus, trends in time cannot be drawn from these data.

For the fatty acids, EPA, and DHA, there were some variations within the same product category for all 3 years (Table 4). As expected, the highest contents of sum n-3, EPA, and DHA were seen in the categories of fatty fish products, where mackerel in tomato sauce had the highest content of all seafood products. However, also the lean fish products such as fish cakes and fish au gratin were good sources of EPA and DHA (Figure 1). The content of vitamin D was generally low in lean seafood products, except for fish cakes in 2017 and 2018 where levels varied between < limit of quantification (LOQ) and 6 μg/100 g (Tables 1b, 2b and 3b). Thus, some products of fish cakes may be considered a source of vitamin D. The highest content of vitamin D was seen in the categories of spread with where salmon/trout had a mean content of 3.8 (±2.3) μg/100 g and 4.8 (±1.0) μg/100 g in 2017 and 2018, respectively. Generally, fatty fish has been recognized as the greatest seafood source of EPA, DHA, and vitamin D. The fatty fish products in this study also had the highest content of these nutrients; however, our data also prove that lean fish products may contribute significantly to the dietary recommended intake of EPA, DHA, and vitamin D if regularly included in the diet. In addition, the lean fish products also contributed with significant amounts of the recommended intake of vitamin B12, iodine, and selenium if regularly consumed.

The largest variation of micronutrients within the same product category were vitamin A and iodine. E.g. the iodine level in fish cakes/burgers varied from 10 to 110 μg/100 g in 2014 (Table 1b) and there was also some variation between years for some products. These variations may be due to different recipes between products, variation of nutrient content in other ingredients, different fish types, as well as natural, geographical, or seasonal variation of nutrients in fish both intra- and inter-species (2, 13). In addition, not all products included in this article declared the amount of fish species used. Iodine has previously been found to vary extensively between different species and even within the same species (15).

Time series of relevant and reliable food composition data of seafood products are important as products may change over years (23, 24). This includes types and amount of fish used and changes in recipes (e.g. type of vegetable oil), in addition to change in other ingredients in the product recipes (e.g. dairy products). Furthermore, the processing of seafood products may change the content and bioavailability of nutrients in fish (25, 26); thus, analysis of raw material of fish may not be sufficient. Also, the consumer habits and preferences of different seafood products may change over time, and new products from the food industry are developed. Therefore, continuous monitoring of nutrients in such products is important to assure reliable food composition data.

### Strengths and limitations

All data presented in this article were analyzed at a national reference laboratory using accredited methods. Accredited laboratory analyses are expensive, and a limited number of analyses are performed for food composition databases (2). The analytic data reported here are therefore an important contribution to the Norwegian food composition database and yield insights into nutrient content of seafood products. The products chosen in this study present a representative list of processed seafood products in Norway. The list includes best-selling products, products from several brands, store brand products, and low-price products, thus representing products available for different consumption patterns, which is a strength of this research. For food composition data, the sampling is fundamental, and several aspects that influence the nutritional content must be taken into consideration in order to acquire high-quality data (4). According to Greenfield and Southgate, the number of sub-samples needed per analytic sample should be calculated from the variance of key nutrients in seafood products in order to achieve averages with reasonable levels of confidence (2). This was not performed in this study; however, most analytic samples consisted of three different batches of which at least three different units were selected from each batch. This is in accordance with most standards, where at least 10 units are used (2, 27, 28), and with the US standards for nutrition labeling which requires 12 units (2). However, composite samples do not allow one to look into the variation within the sample, which is a limitation.

## Conclusion

In this article, we have described the nutrient concentrations in commercially available seafood products in Norway, collected from three different years. The amount of fish and species used in processed seafood were variable. We found that several processed seafood products may contribute significantly to the RI for several essential nutrients, such as EPA, DHA, vitamin D, vitamin B12, iodine and selenium, if included in the diet. Both the type of fish and the amount of fish used influenced the nutrient contents. Some variations in nutrient content between years and within products were found, and we cannot conclude on whether this was caused by natural variations, variations along the value chain, or variations in nutrient content of the fish. Thus, it is of importance to monitor food composition of seafood products to assess time trends. Relevant, reliable, and up-to-date food composition data are important from several points: from nutrient intake estimations in the clinic to accuracy in nutrition research and all the way up to policymaking on nutrition and agriculture.

## Supplementary Material

New data on nutrient composition in large selection of commercially available seafood products and its impact on micronutrient intake
