# Supplementary material for: New data on nutrient composition in large selection of commercially available seafood products and its impact on micronutrient intake
Source: Food Nutr Res. 2019 Jul 8;63:10.29219/fnr.v63.3573. doi: 10.29219/fnr.v63.3573 (PMC6642616; doi:10.29219/fnr.v63.3573)
Supplement: New data on nutrient composition in large selection of commercially available seafood products and its impact on micronutrient intake [file FNR-63-3573-s001.pdf]

Supplementary material

Supplemental 1 Description of the different seafood products including content of fish (%), fish species and number of seafood products analyzed each year.

**Seafood product**	**Total fish content (%)**[a]	**Fish description (specific fish type (%))**[a]	**Number of seafood products**[b]		
			2015 (n=16)	2017 (n=35)	2018 (n=35)
**Fish cakes/burgers**					
‘Berggren’ fish burgers	50%	Greater argentine whole fish (*Argentina silus*); Surimi (*Argentina silus*). % of specific species not given.	x	x	
‘Best pris’ fish cakes	53%	Greater argentine whole fish (*Argentina silus*)			x
‘Coop’ fish cakes	2015: 60%	Greater argentine whole fish (*Argentina silus*) (39%); Haddock fillet (*Melanogrammus aeglefinus*) (21%);	x	x	x
	2017, 2018: 65%	Greater argentine whole fish (*Argentina silus*) (31%); Haddock fillet (*Melanogrammus aeglefinus*) (18%); Alaska pollock (*Theragra chalcogramma*) (% not given); Pacific whiting (*Merluccius productus*) (% not given)			
‘First Price’ fish cakes	52%	Greater argentine whole fish (*Argentina silus*); Alaska pollock (*Theragra chalcogramma*); Haddock fillet (*Melanogrammus aeglefinus*); Saithe (*Pollachius virens*); Cod fillet (*Gadus morhua*). % of specific species not given.		x	x
‘Fiskemannen’ fish cakes	2015, 2017: 65%	2015, 2017: Greater argentine whole fish (*Argentina silus*); Saithe (*Pollachius virens*); Haddock fillet (*Melanogrammus aeglefinus*). % of specific species not given.	x	x	x
	2018: 80%	2018: Greater argentine whole fish (*Argentina silus*) (46%); Haddock fillet (*Melanogrammus aeglefinus*) (60%); Cod fillet (*Gadus morhua*) (10%)			
‘Fiskemannen’ fish burgers	2015: 70%	Greater argentine whole fish (*Argentina silus*); Haddock fillet (*Melanogrammus aeglefinus*). % of specific species not given.	x	x	x
	2017, 2018: 80%	2017: Greater argentine whole fish (*Argentina silus*) (53%); Cod fillet (*Gadus morhua*) (14%); Haddock fillet (*Melanogrammus aeglefinus*) (13%)			
		2018: Greater argentine whole fish (*Argentina silus)* (50%); Cod fillet (*Gadus morhua*) (19%); Haddock fillet (*Melanogrammus aeglefinus*) (11%)			
‘Godehav’ fish cakes	2015: 58%	2015: Greater argentine whole fish (*Argentina silus*)	x	x	

	2017: 70%	2017: Greater argentine whole fish (*Argentina silus*) (49%); Haddock fillet (*Melanogrammus aeglefinus*) (21%)			
‘Godehav’ fish burgers	2015: 50%<br>2017: 88%	Greater argentine whole fish (*Argentina silus*)<br>Haddock fillet (*Melanogrammus aeglefinus*); Cod fillet (*Gadus morhua*). % of specific fish species not given.	x	x	
‘Lofoten’ fish burgers	86%	Haddock fillet (*Melanogrammus aeglefinus*) (43%), Cod fillet (*Gadus morhua*) (22.5%); Greater argentine whole fish (*Argentina silus*) (20.5%)			x
‘Lofoten’ homemade fish cakes	60%	Haddock fillet (*Melanogrammus aeglefinus*)	x	x	x
‘X-tra’ fish cakes	2018: 56%<br>2017: 57%	Greater argentine whole fish (*Argentina silus*) (37%); Haddock fillet (*Melanogrammus aeglefinus*) (19%)<br>Greater argentine whole fish (*Argentina silus)* (26%); Haddock fillet (*Melanogrammus aeglefinus*) (14%); Surimi (Alaska pollock, *Theragra chalacogramma*) (17%)		x	x
‘Rema 1000’ fish burgers XXL	50%	Greater argentine whole fish (*Argentina silus*)			x
**Fish au gratin**					
‘X-tra’ fish au gratin	2015: 25%<br>2017, 2018: 30%	Saithe fillet (*Pollachius virens*)	x	x	x
‘Enghav’ fish au gratin	25%	Saithe fillet (*Pollachius virens*)	x		
‘Findus’ ‘Familiens’ fish au gratin	24%	Alaska pollock (*Theragra chalcogramma*)	x	x	x
‘Findus’ ‘God Gammeldags’ fish au gratin	30%	Cod fillet (*Gadus morhua*)		x	x
‘First Price’ fish au gratin	30%	Saithe fillet (*Pollachius virens*)	x	x	x
‘ICA’ fish au gratin	20%	Saithe fillet (*Pollachius virens*)	x		
**Fish fingers/panned fish products**					
‘Coop’ fish fingers	66%	Cod fillet (*Gadus morhua*)		x	
‘Findus’ fish fingers	61%	Alaska pollock (*Theragra chalcogramma*)	x	x	x
‘First Price’ fish fingers	59%	Alaska pollock (*Theragra chalcogramma*)		x	x
‘Lerøy’ fish fingers	65%	Cod fillet (*Gadus morhua*)		x	x
‘X-tra’ fish fingers	60%	Cod fillet (*Gadus morhua*)			x
‘Findus’ panned cod fillet	2015: 64%<br>2017, 2018: 65%	Cod fillet (*Gadus morhua*)	x	x	x
**Saithe products**					
‘First Price’ panned saithe fillet	65%	Saithe fillet (*Pollachius virens*)	x	x	x

‘Lerøy’ breaded saithe fillet	70%	Saithe fillet (*Pollachius virens*)	x	
**Fish pudding**				
‘First Price’ fish pudding	52%	Greater argentine whole fish (*Argentina silus*); Alaska pollock (*Theragra chalcogramma*); Haddock fillet (*Melanogrammus aeglefinus*). % of specific species not given.	x	x
‘Fiskemannen’ fish pudding	56%	Greater argentine whole fish (*Argentina silus*); Alaska pollock (*Theragra chalcogramma*); Haddock fillet (*Melanogrammus aeglefinus).* % of specific species not given.	x	x
‘Best pris’ fish pudding	53%	Greater argentine whole fish (*Argentina silus*)		x
‘Godehav’ fish pudding	58%	Greater argentine whole fish (*Argentina silus*) (53%); Haddock fillet (*Melanogrammus aeglefinus*) (5%)	x	
**Spread - salmon/trout**				
‘Fiskemannen’ smoked salmon	100%	Atlantic salmon fillet (farmed) (*Salmo salar*)	x	x
‘Godehav’ smoked trout	100%	Rainbow trout fillet (farmed) (*Oncorhynchus mykiss*)	x	x
‘Lerøy’ smoked salmon	100%	Atlantic salmon fillet (farmed) (*Salmo salar*)	x	x
‘Lerøy’ hot-smoked trout	92.5%	Rainbow trout fillet (farmed) (*Oncorhynchus mykiss*)		x
‘Lerøy’ smoked trout	100%	Rainbow trout fillet (farmed) (*Oncorhynchus mykiss*)	x	
‘Lofoten’ smoked salmon	100%	Atlantic salmon fillet (farmed) (*Salmo salar*)	x	x
‘Stabburet’ hot-smoked salmon	2017: 70%<br>2018: 75%	Atlantic salmon fillet (farmed) (*Salmo salar*)	x	x
**Spread - mackerel in tomato sauce**				
‘Coop’ mackerel in tomato sauce	70%	Mackerel fillet (*Scomber scombrus*)	x	x
‘King Oscar’ mackerel in tomato sauce	2017: 50%<br>2018: 62%	Mackerel fillet (*Scomber scombrus*)	x	x
‘Stabburet’ mackerel in tomato sauce	2017: 70%<br>2018: 60%	Mackerel fillet (*Scomber scombrus*)	x	x
**Spread - caviar**				
‘First Price’ caviar	52%	Roe from cod (*Gadus morhua*) and saithe (*Pollachous virens*)	x	x
‘Kavli’ caviar	60%	Cod roe (*Gadus morhua*)	x	x
‘Mills’ caviar	45%	Cod roe (*Gadus morhua*)	x	x
‘Rema 1000’ caviar	52%	Roe from cod (*Gadus morhua*) and saithe (*Pollachous virens*)	x	x

[a] If different fish content or fish species in products between years this is specified for the specific product. [b] Each seafood product consists of one composite sample, comprising three different batches where four different packages were included in each batch
